# Biomass Pyrolysis Solids as Reducing Agents: Comparison with Commercial Reducing Agents

**DOI:** 10.3390/ma9010003

**Published:** 2015-12-23

**Authors:** Aitziber Adrados, Isabel De Marco, Alexander López-Urionabarrenechea, Jon Solar, Blanca M. Caballero, Naia Gastelu

**Affiliations:** Department of Environmental and Chemical Engineering, University of the Basque Country UPV/EHU, Bilbao 48013, Spain; isabel.demarco@ehu.eus (I.D.M.); alex.lopez@ehu.eus (A.L.-U.); jon.solar@ehu.eus (J.S.); blancamaria.caballero@ehu.eus (B.M.C.); naia.gastelu@ehu.eus (N.G.)

**Keywords:** biomass, biocoke, bioreducer, slow pyrolysis

## Abstract

Biomass is one of the most suitable options to be used as renewable energy source due to its extensive availability and its contribution to reduce greenhouse gas emissions. Pyrolysis of lignocellulosic biomass under appropriate conditions (slow heating rate and high temperatures) can produce a quality solid product, which could be applicable to several metallurgical processes as reducing agent (biocoke or bioreducer). Two woody biomass samples (olives and eucalyptus) were pyrolyzed to produce biocoke. These biocokes were characterized by means of proximate and ultimate analysis, real density, specific surface area, and porosity and were compared with three commercial reducing agents. Finally, reactivity tests were performed both with the biocokes and with the commercial reducing agents. Bioreducers have lower ash and sulfur contents than commercial reducers, higher surface area and porosity, and consequently, much higher reactivity. Bioreducers are not appropriate to be used as top burden in blast furnaces, but they can be used as fuel and reducing agent either tuyére injected at the lower part of the blast furnace or in non-ferrous metallurgical processes where no mechanical strength is needed as, for example, in rotary kilns.

## 1. Introduction

The metallurgical industry is a great consumer of fossil fuels. It uses great amounts of coke as fuel and reducing agent [1,2,3], with the consequence of large emissions of carbon dioxide (CO_2_), the primary greenhouse gas emitted by human activities [4].

International concerns about climate change are leading to a decrease in CO_2_ emissions. Recently, the Intergovernmental Panel on Climate Change (IPCC) identified in the Fifth Assessment Report the world’s carbon budget [5], *i.e.*, the amount of CO_2_ the world can emit while still having a likely chance of limiting global temperature rise to 2 °C above pre-industrial levels. The 2 °C target was adopted by the countries within the United Nations Framework Convention on Climate Change (UNFCC) [6]. The international scientific community estimates this budget to be 1 trillion tons of carbon. In 2011, 52% of the budget had already been burned, and it is estimated that the budget will be exceeded before the end of 2045 if emissions rates continue with the same tendency [5]. To avoid this, new ideas and concepts must be developed by the industry.

The production of reducing agents (biocokes or bioreducers) by pyrolysis of biomass is one of these ideas and a step further in the reduction of the environmental impact of industry.

The key factor in the production of coke from biomass is the fact that biomass derived products and processes imply neutral CO_2_ emissions. Biomass absorbs atmospheric CO_2_ while it grows and returns it into the atmosphere when it is burned, all in a relatively short amount of time. Because of this, biomass utilization creates a closed loop carbon cycle. The substitution of fossil fuel coke by more environmentally friendly biocokes is of the most interest. For this reason, the objective of this paper is to compare the properties of biocokes obtained by pyrolysis of lignocellulosic biomass with those of typical commercial reducing agents used in non-ferrous processes, in order to evaluate the suitability of the biocokes as reducing agents.

The production of biocoke from biomass is not new; in fact it is the update and modernization of the traditional process of obtaining charcoal from wood. In recent years the effects of the type of biomass and of the operating parameters used on the properties of the obtained char have been investigated [7,8,9,10,11,12].

The production of a coke-like product from biomass requires more demanding process conditions than the traditional method, since traditional coke is a macroporous carbon material produced by carbonization of coals or coal blends of specific rank and characteristics [13]. In other words, to obtain a coke-like product from wood, the carbonization of wood followed by the carbonization of the resultant coal must occur in the same process. Griessacher and Antrekowitsch [14] and Griessacher, *et al.* [15] reported that a coke-like product can be obtained by slow pyrolysis of wood at high temperature (700–1000 °C).

One of the most important influencing parameter of the slow pyrolysis (carbonization) process is temperature. It is well known that by increasing temperature the amount of volatile components in the coal decreases and therefore the carbon content in the coal becomes higher reaching the carbon content of cokes [16]. Heating rate is also a very important parameter that influences the amount and characteristics of pyrolysis products. It has been reported [17] that a higher char yield is obtained with decreasing heating rate, since the slow emerging wood products have more time to react and carbonize. It may be expected that the lower the heating rate, the lower the char porosity and reactivity since the volatile products from the biomass emerge more slowly. The objective of the present work was to obtain solids from biomass useful as reducing agents for metallurgical processes (biocoke or bioreducers). Therefore, high temperatures and slow heating rates were used. On the other hand, the feedstock material is a key factor for the production of biocoke, and therefore, two woody biomass samples, olive tree cuttings, and eucalyptus, were used in this study. It is well known that woody biomass is the best biomass feedstock for coke production.

In order to evaluate the suitability of the pyrolysis solids as alternative reducing agents in metallurgy, it is important to characterize the biocokes, by determining their chemical and physical properties, and also their reactivity with CO_2_. There are standardized reactivity measurement methods like the ASTM D 5341-99 standard [18,19] which was developed from the Nippon Steel Corporation (NSC) test procedure, and has been traditionally used for evaluating the quality of coal derived metallurgical cokes.

In this study, characterization and reactivity measurements of the biocokes obtained in pyrolysis experiments as well as of three commercial reducing agents used in metallurgical processes were carried out.

## 2. Materials and Methods

### 2.1. Materials

Two different biomass samples were pyrolyzed: an abundant waste woody biomass sample, olive tree cuttings, coming from the South of Spain and an extensive energy crop, eucalyptus (camaldulensis) wood, coming from South America.

The olive tree cuttings sample was selected since the major woody crop in Spain is olive. In Spain there are 2,605,252 ha of olive groves [20]. One hectare of olive groves generates about three tons of pruning residues [21] which means 7,815,756 tons of branches and leaves per year, most of which are illegally burnt or left on the ground partially for recirculation of nutrients.

On the other hand, the eucalyptus family was selected as an extensive energy crop because of its fast growth rate and abundance, especially in South America. It is worth mentioning that Eucalyptus occupies 70% of the 950,000 ha of the forest plantation in Uruguay and 60% of the 5,500,000 ha of forest plantation in Brazil.

The composition of both biomass samples is presented in Table 1.

**Table 1:** Proximate, ultimate and constituents analyses of the original pyrolyzed biomass samples.

(Weight %)		Olives	Eucalyptus
Proximate analysis (ar) ^a^	Moisture	8.8	11.4
	Volatile matter	74.3	69.7
	Ash	2.1	2.1
	Fixed carbon ^c^	14.8	16.8
Ultimate analysis (daf) ^b^	C	49.4	52.7
	H	6.2	3.7
	N	0.3	0.1
	S	<0.1	<0.1
	Others (mainly O) ^c^	44.1	43.5
HHV (ar) ^a^ (MJ·kg^−1^)		16.1	16.6
H/C atomic ratio		1.5	0.8
C/N weight ratio		146.7	455.0
Constituents (daf) ^b^	Extractives	12.1	8.3
	Cellulose	32.9	38.5
	Hemicellulose	19.7	13.6
	Lignin	18.3	29.3
	Others ^c^	15.7	10.3

^a^ ar: as received basis; ^b^ daf: dry and ash free basis; ^c^ By difference.

It can be observed that the eucalyptus and the olive tree cuttings samples are quite different. The eucalyptus sample has more moisture, less volatiles and more fixed carbon than the olive sample. Concerning the ultimate analysis, the eucalyptus sample has more C%, less H% and less N% than the olives sample, and as a consequence has lower H/C and higher C/N ratios than the olives sample. The lower the C/N ratio, the higher the degradability of the organic matter, so the results indicate that eucalyptus is more resistant to biodegradation than the olives sample.

Concerning the constituents’ composition, Table 1 shows that the eucalyptus sample has greater lignin content and lower hemicellulose content than the olives sample. With respect to extractives, it has been reported that they are more abundant in bark than in inner wood parts [22]. This justifies the fact that the eucalyptus sample, which has a lower bark/wood ratio than the olives sample, has less extractives.

Regarding the higher heating value of the biomass samples, there are no significant differences between them. Both values are around 16.4 MJ·kg^−1^, which are heating values comparable to those of low quality fuels like lignites (≈16 MJ·kg^−1^).

In order to compare the properties of the pyrolitically produced biocokes, three commercial reducers provided by Befesa Zinc Aser S.A., an Electric Arc Furnace dust recycling company, located in Erandio (Biscay, Spain) were analyzed and tested: a metallurgical coke, a petroleum coke, and an anthracite. The characterization of these commercial reducers is presented in Table 5, and is discussed and compared to biocoke characteristics in Section 3.1. 

### 2.2. Experimental Procedure

#### 2.2.1. Pyrolysis Experiments

Pyrolysis experiments with biomass samples were carried out using two reactors connected in series: a first 3.5 L semi-batch non-stirred pyrolysis reactor where 100 g of biomass were pyrolyzed at 600 °C and 750 °C and with heating rates of 3, 15, and 20 °C·min^−1^, and a second tubular reactor where pyrolysis vapors were thermally upgraded at 900 °C. The vapors were swept with 1 L·min^−1^ of nitrogen (N_2_) to a water cooled glass condensing system, where liquids were collected, followed by an activated carbon column (to retain the possible small particles that the gases could transport) before they were collected in plastic gas bags. The biocokes produced remained in the first reactor. The solid and liquid yields were determined by weight difference, while gas yield was calculated by difference to 100. Each yield presented in this paper is the mean value of the data obtained in two equivalent experiments which differed by less than three points. A flow sheet of the experimental setup used can be seen in Adrados, *et al.* [23]. 

#### 2.2.2. Reactivity Experiments

The procedure used for the reactivity experiments is based on the ASTM D 5341-99 standard [18]. However, to meet the specifications of this standard, specific large equipment and a great amount of sample (250 g) is required. Since neither of them was available, an adaptation of the ASTM method had to be carried out trying to keep the different operational parameters as close as possible to the specifications of the standard. This ASTM standard is based on the Boudouard reaction ($\text{C}+{\text{ CO}}_{2}↔\text{CO}$), in which carbon reacts with CO_2_ and forms carbon monoxide (CO). Depending on the reactivity of the material, the quantity of carbon consumed is variable.

Two parameters were determined in the reactivity tests: the so called coke reactivity index (CRI) and the *R* factor (1) and (2).

(1) $$\text{CRI }\left(\%\right)=\frac{\text{initial sample weight }–\text{ final weight}}{\text{initial sample weight}}\times 100$$

(2) R factor (%)=COCO2+CO2×100

The CRI is the mass weight loss after the treatment of the sample with CO_2_ or the degree of conversion (wt %) and the R factor is the amount of CO in the gas stream after reaction with respect to the total amount of CO_2_ used in the test (vol %). High values of both parameters indicate high reactivity of the sample.

The reactivity experiments with the biocokes and the commercial reducers were performed in the same plant used for pyrolysis experiments but adapted for the reactivity tests. In this case, the installation was composed of four units connected in series: (1) a first 3.5 L empty preheater, where the feed gases were pre-heated to 800 °C; (2) a second tubular reactor where a fixed bed of the biocoke was placed; (3) a dust trap (empty bubbler) where the small solid particles swept by the CO_2_ flow were retained; and (4) Tedlar plastic bags where the gases of the whole test were collected. All the apparatus were connected with silicone tubing.

The tubular reactor used for the reactivity experiments was 2.54 cm in diameter and 60 cm long, and was made of INCONEL alloy 601, a material able to work at temperatures as high as 1200 °C, while in the pyrolysis experiments a 309 stainless steel tubular reactor was used.

The system was purged with N_2_ in order to remove the oxygen (O_2_) originally contained in the installation. The reactor’s furnace was warmed up to 1000 °C, and then a CO_2_ gas flow of 1375 or 750 mL·min^−1^ was passed through the sample for 2 h. The charcoal reacts with CO_2_, producing CO. The gases generated in the whole run were continuously collected in plastic bags, to be immediately analyzed off-line with a gas chromatograph connected to a thermal conductivity and a flame ionization detector (GC-TCD, FID). After 2 h, the input gas stream was again swapped to N_2_ and the furnace was cooled down.

### 2.3. Analytical Techniques

The initial feedstock, the solid pyrolysis products, and the commercial reducers were characterized using the following analytical techniques. The proximate analysis was determined by thermogravimetry according to D3173-85 and D3174-82 ASTM standards by means of a LECO TGA-500, and the elemental composition was determined with a LECO TruSpec CHN and a TruSpec S analyzer. The higher heating value (HHV) was determined using a LECO AC-500 automatic calorimetric bomb.

Real density was determined in an AccuPyc 1330T Micromeritics equipment. All the samples were degassed at 120 °C for 18 h before the analyses. The surface areas and the porosity properties were measured via CO_2_ adsorption at 0 °C using a surface area analyzer, Quantachrome Nova 4200 apparatus. CO_2_ adsorption is a widely used method to analyze materials with narrow micropores, as is the case of carbonized materials. The isotherms were analyzed using the Dubinin Radushkevich equation (DR) for calculating the micropores volume (cm^3^·g^−1^) and the equivalent micropore surface area (m^2^·g^−1^). The pore size distribution was calculated using the Non Local Density Functional Theory (NL DFT) available in the software of the equipment.

## 3. Results and Discussion

### 3.1. Yields, and Proximate and Ultimate Analysis of Pyrolysis Solids

The characteristics of the solids obtained in the pyrolysis experiments can be influenced by the operating conditions of the first pyrolysis reactor. Olive tree cuttings and eucalyptus were pyrolyzed at different temperatures and heating rates, therefore, several biochars were obtained. Their yields and characterization are presented in Table 2 and Table 3.

**Table 2:** Proximate and ultimate analyses of the pyrolysis solids obtained at different temperatures (20 °C·min^−1^).

(Weight %)		Olives		Eucalyptus	
		600 °C	750 °C	600 °C	750 °C
Solid yields		22.9	21.3	22.4	22.9
Proximate analysis (ap) ^a^	Moisture	2.0	1.7	2.2	1.4
	Volatile matter	11.8	10.0	12.0	8.6
	Ash	9.5	9.2	5.9	6.2
	Fixed Carbon ^c^	76.7	79.1	79.9	83.8
Ultimate analysis (daf) ^b^	C	86.0	94.5	88.0	90.1
	H	2.1	0.9	1.4	0.8
	N	0.8	1.2	0.6	0.6
	Others (mainly O) ^c^	11.1	3.4	10.0	8.5
HHV (ap) ^a^ (MJ kg^−1^)		30.8	28.8	31.6	31.0

^a^ ap: as produced basis; ^b^ daf: dry and ash free basis; ^c^ By difference.

**Table 3:** Characteristics of the pyrolysis solids obtained at different heating rates (750 °C).

(Weight %)		Olives			Eucalyptus	
		20 °C·min^−1^	15 °C·min^−1^	3 °C·min^−1^	20 °C·min^−1^	3 °C·min^−1^
Solid yields		21.3	24.6	26.1	22.9	26.0
Proximate analysis (ap) ^a^	Moisture	1.7	1.6	1.7	1.4	1.4
	Volatile matter	10.0	10.1	10.6	8.6	8.0
	Ash	9.2	10.2	9.2	6.2	5.3
	Fixed Carbon ^b^	79.1	78.1	78.5	83.8	85.3
Ultimate analysis (daf) ^c^	C	94.5	92.9	94.6	90.1	96.2
	H	0.9	1.1	0.8	0.8	0.9
	N	1.2	1.4	1.3	0.6	0.9
	Others (mainly O) ^b^	3.4	4.6	3.3	8.5	2.0
HHV (ap) ^a^ (MJ kg^−1^)		28.8	27.5	29.5	31.0	31.6

^a^ ap: as produced basis; ^b^ By difference; ^c^ daf: dry and ash free basis.

Taking a look at the solid yields, in Table 2 it can be seen that temperature has not much influence. However, Table 3 shows an influence of the heating rate: the lower the heating rate, the higher the solid yield. These results are in agreement with the results obtained by other authors [24,25]. The pyrolysis solid yields are in the range of 21–23 wt % at 20 °C·min^−1^ and 26 wt % when the lowest heating rate is used. Therefore the reduction of heating rate has the beneficial effect of increasing the pyrolysis solids yields. Liquid and gas yields obtained in these pyrolysis experiments can be found in [26].

Pyrolysis solids are mainly composed of carbon, with elemental carbon contents around 90 wt %. The moisture content is rather low (≈1.7 wt %), the volatiles content is quite low (≈11 wt %) and the ash content is around 9 and 6 wt % for olives and eucalyptus samples respectively.

It is well known that the higher the fixed carbon and the lower the volatile matter content, the better is the quality of metallurgical reducing agents. Table 2 shows that the fixed and elemental carbon contents of the biocokes of both samples are higher while the volatile matter is lower when the higher pyrolysis temperature (750 °C) is used. Similar tendencies regarding the effect of temperature in the fixed carbon and volatile matter contents of pyrolysis solids have been reported in the literature [27].

Table 3 shows that there is no influence of heating rate on the composition of pyrolysis solids. Although it is a well-known fact that decreasing heating rates intensifies the carbonization process giving rise to carbon-richer products, this tendency is not observed in the results of Table 3. The reason for this may be that 20 °C·min^−1^ is a heating rate slow enough to produce a well carbonized product at 750 °C, so that slower heating rates do not further promote carbonization at such temperature.

Regarding the HHV of the pyrolysis solids it can be seen that it is rather high (≈30 MJ·kg^−1^) which is in the range of the HHV of commonly used solid fossil fuels, such as bituminous coals [28].

In view of the characteristics of biomass derived pyrolysis solids, many potential applications for them can be proposed. They can be used as rather good quality solid fuel (high HHV, low pollutants (S, N) content and low ash contents compared to fossil fuels), or as sorbent material provided that it is firstly activated, or as soil amendment agent. However, the application proposed in this work is as reducing agent for metallurgical purposes. For this reason, the three previously mentioned commercial reducing agents (metallurgical coke, petroleum coke, and anthracite), used for Zn reduction in rotary kilns by Befesa Zinc Aser S.A. company, were characterized. The detailed specifications that Befesa Zinc Aser S.A. requires from their providers of reducing agents are presented in Table 4. The specifications on dry basis, which are also included in the table, have been calculated considering that the moisture content is 20 wt %, which is as the table shows, the maximum allowable moisture specified by Befesa; these data on dry basis would be the permitted values if the sample did not contain moisture and enable coke samples to be compared regardless of their moisture.

**Table 4:** Quality specifications for commercial reducing agents required by Befesa Zinc Aser S.A. company 2013.

Material	Parameter	Befesa Zinc Aser Technical Specifications (wt %)	Specifications on Dry Basis (wt %) *
Metallurgical coke	Granulometry	>10 mm: ≤20% <2 mm: ≤40% on daily sample	–
	Dry ash	≤20% on monthly sample	≤20%
	Moisture	≤20% on daily sample	–
	Volatile matter	≤7% on monthly sample	≤8.75%
	Sulfur	≤3% on monthly sample	≤3.75%
Petroleum coke	Granulometry	<2 mm: >30% >10 mm: ≤20% on diary sample	–
	Dry ash	≤20% on monthly sample	≤20%
	Moisture	≤20% on daily sample	–
	Volatile matter	≤15% on monthly sample	≤18.75%
	Sulfur	≤3% on monthly sample	≤3.75%
Anthracite	Granulometry	<2 mm: >30% >10 mm: ≤20% on diary sample	–
	Dry ash	≤20% on monthly sample	≤20%
	Moisture	≤20% on daily sample	–
	Volatile matter	≤7% on monthly sample	≤8.75%
	Sulfur	≤3% on monthly sample	≤3.75%

* Calculated considering a moisture content of 20 wt % (maximum permitted by Befesa).

Table 5 presents the proximate and ultimate analyses of the commercial reducing agents and the biomass-based reducing agents obtained at 3 °C·min^−1^ and 750 °C, all on dry basis. For better analysis of the results, the Befesa specifications are included in brackets in Table 5.

**Table 5:** Proximate and ultimate analyses of the commercial reducers and the bioreducers (750 °C and 3 °C·min^−1^).

Dry Basis (wt %)		Commercial Reducers			Bioreducers	
		Metallurgical Coke	Petroleum Coke	Anthracite	Olives	Eucalyp.
Moisture		11.4 (<*20*)	6.4 (<*20*)	18.0 (<*20*)	1.7	1.4
Proximate analysis	Volatile matter	3.9 (<*8.75*)	10.1 (<*18.75*)	7.2 (<*8.75*)	10.8	8.1
	Ash	12.5 (<*20*)	2.0 (<*20*)	11.2 (<*20*)	9.4	5.4
	Fixed carbon *	83.6	87.9	81.6	79.9	86.5
Ultimate analysis	C	84.4	83.5	86.6	85.6	90.9
	H	0.5	2.8	0.6	0.8	1.0
	N	1.0	1.3	1.0	1.2	0.8
	S	0.9 (<*3.75*)	5.6 (<*3.75*)	0.6 (<*3.75*)	<0.05	<0.05
	Others *	0.8	4.9	0.0	3.0	1.9
HHV (MJ·kg^−1^)		29.3	35.9	31.1	29.5	31.6

* By difference; ( ) In brackets Befesa specifications on dry basis.

It is a fact worth mentioning that the commercial reducers have very high moisture contents. Such contents certainly do not correspond to the natural inherent moisture of these materials, and it is most probably accidental moisture incorporated into the samples during their transport and/or storage. As a matter of fact, water drops could be seen in the containers in which the commercial reducers were provided. For this reason, the proximate and ultimate analyses of all the samples are presented in Table 5 on dry basis, in order to more fairly compare the intrinsic properties of the commercial and the biomass derived reducers, regardless of the moisture content.

It has also to be mentioned that although the commercial reducers were provided by Befesa Zinc Aser S.A. itself, one of them (petroleum coke) does not totally fulfill the quality requirements specified by the company, since it contains more sulfur (5.6 wt %) than that specified in Table 4 (<3 wt %).

Comparing the results of the commercial reducers and the bioreducers, the following advantages of the bioreducers can be mentioned: they have much lower moisture contents than any of the commercial reducers, lower ash contents than the metallurgical coke and the anthracite, and significantly lower sulfur contents than any of the commercial reducers. There is only one specification not fulfilled in the bioreducer derived from the olives sample: the volatile content is slightly higher than that specified for metallurgical coke and anthracite, though it does meet, by far, the petroleum coke volatile matter specification.

Therefore, it can be concluded that as far as composition is concerned, the olives and eucalyptus derived bioreducers can replace the commercial reducers in metallurgical processes in rotary kilns with the great advantage of having lower ash and sulfur contents.

### 3.2. Real Density, Specific Surface Area, and Porosity Measurements

Real density, specific surface area, and porosity of the pyrolysis reducers and of the commercial reducers were measured.

**Figure 1:**
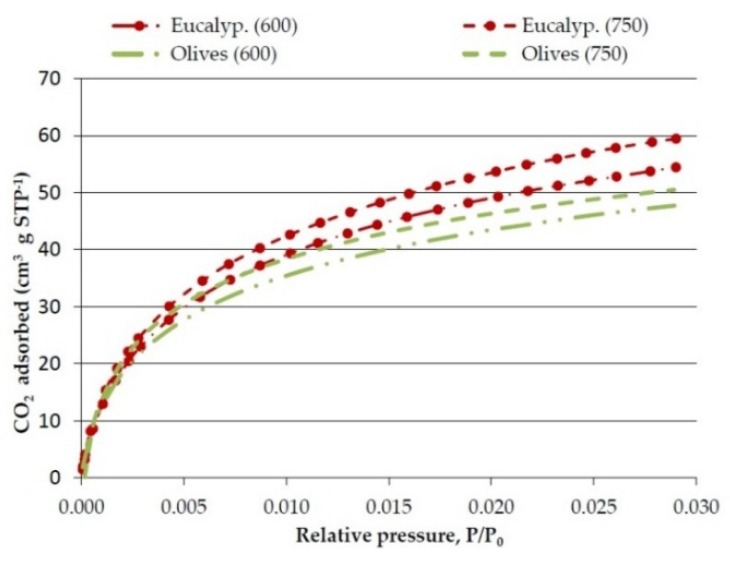
Effect of temperature on CO_2_ adsorption isotherms of the bioreducers obtained at 20 °C·min^−1^ from olives and eucalyptus samples.

**Figure 2:**
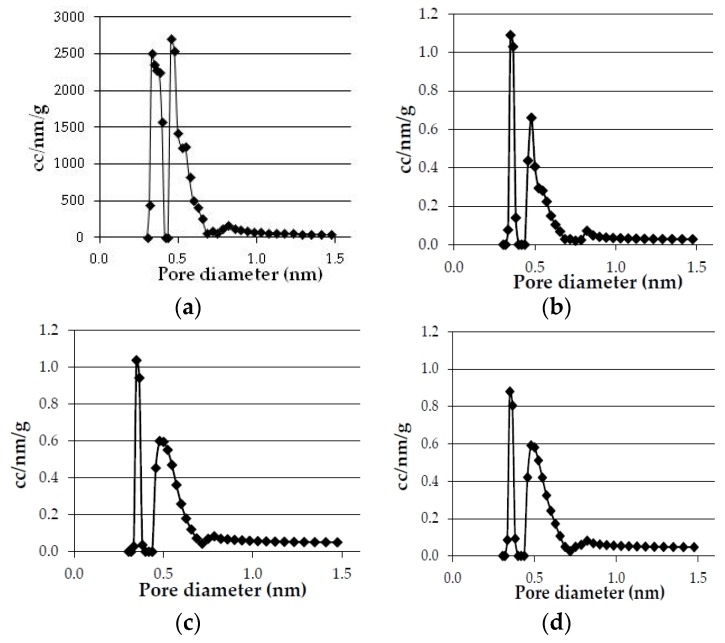
Effect of temperature in pore size distribution of the bioreducers obtained at 20 °C·min^−1^ from olives and eucalyptus samples. (**a**) Olives (750 °C); (**b**) Olives (600 °C); (**c**) Eucalyptus (750 °C); (**a**) Eucalyptus (600 °C).

The results obtained in the textural characterization of the pyrolysis solids obtained at 20 °C·min^−1^ and different pyrolysis temperatures are presented in Figure 1 and Figure 2, and Table 6.

**Table 6:** Effect of the temperature in the textural characterization of the bioreducers (20 °C·min^−1^).

	Temperature (°C)	Real Density (g·cm^−3^)	Micropore Volume (cm^3^·g^−1^)	Micropore Equivalent Surface Area (m^2^·g^−1^)
Olives	600	1.669	0.15	360
	750	1.926	0.16	375
Eucalyptus	600	1.622	0.19	444
	750	1.849	0.20	476

The CO_2_ adsorption isotherms presented in Figure 1 show the CO_2_ volume adsorbed (cm^3^·g^−1^) against the relative pressure (*P*/*P*_0_). The CO_2_ adsorption isotherms are clearly of Type I that are typical of microporous materials. Although there is some controversy, it is frequently considered that the micropores volume determined by CO_2_ adsorption corresponds to narrow micropores (<0.7 nm).

The pore size distributions of the bioreducers samples calculated by the NL-DFT method are presented in Figure 2, which show that the bioreducers do not have micropores greater than 0.7 nm, therefore, the micropores volume determined corresponds to the total pores volume.

Concerning the effect of the pyrolysis temperature, Table 6 shows that the real density clearly increases as the temperature is raised from 600 °C to 750 °C, while the micropore volume and surface area also increases but very slightly.

The increase of micropore surface area with temperature is contrary to what has been observed by Burhenne, *et al.* [29] who reported a decrease in char surface area when the pyrolysis temperature was raised from 500 °C to 800 °C. This discrepancy may be due to differences in the surface area determination methods: N_2_ (used by Burhenne) *versus* CO_2_ adsorption (used in this study). Burhenne’s reasoning is that at higher temperature an occlusion of most micropores occurs; however it is plausible that what happens is that at higher pyrolysis temperature, narrower micropores are produced, and these, as will be explained next, are difficult to be measured with liquid N_2_.

Agirre, *et al.* [30] also found that the surface area increases when the pyrolysis temperature is raised. The reason for the increase of surface area with temperature may be that at higher temperatures greater amount of volatiles are driven out of the solid, giving rise to more micropores.

**Figure 3:**
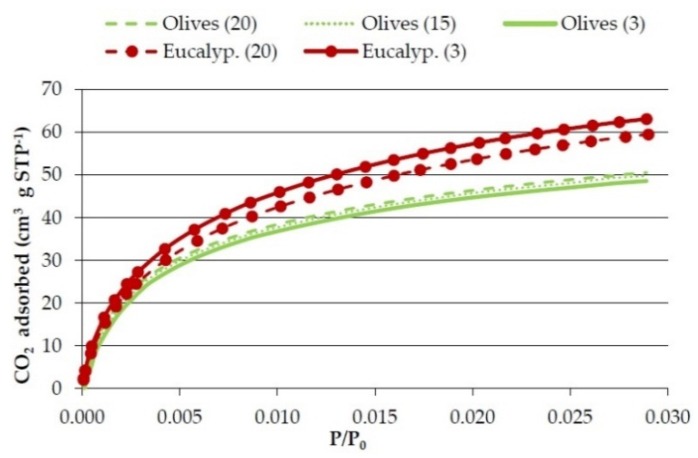
Effect of heating rate on CO_2_ adsorption isotherms of the bioreducers obtained at 750 °C from the olives and eucalyptus samples.

**Figure 4:**
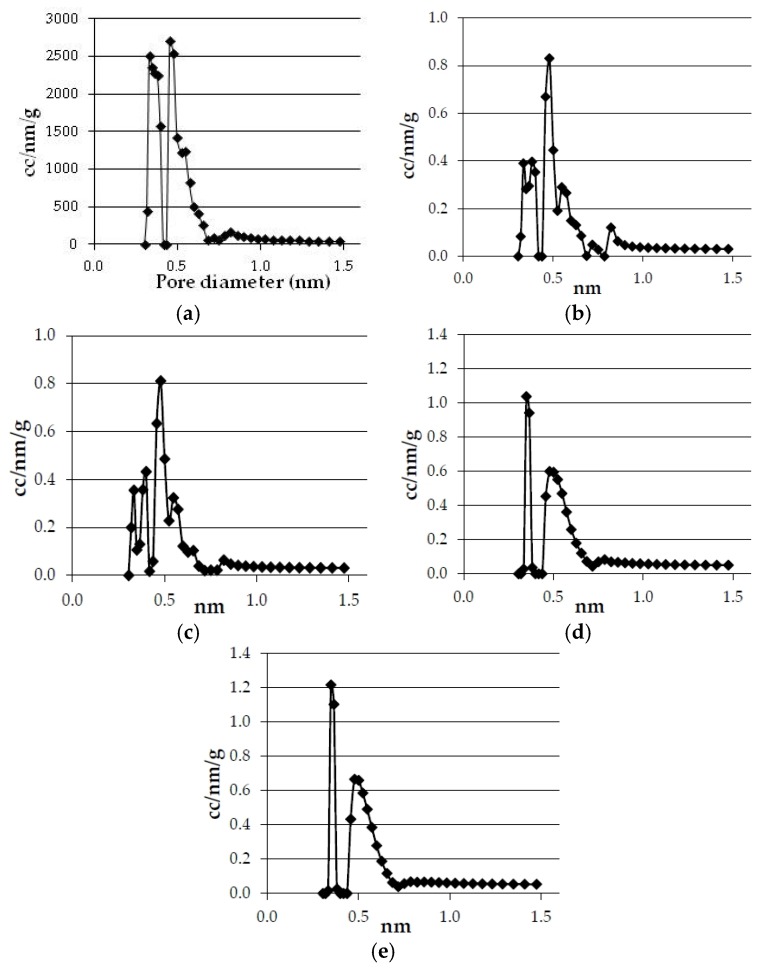
Effect of heating rate in pore size distribution of the bioreducers obtained at 750 °C from the olives and eucalyptus samples. (**a**) Olives 20 °C·min^−1^; (**b**) Olives 15 °C·min^−1^; (**c**) Olives 3 °C·min^−1^; (**d**) Eucalyptus 20 °C·min^−1^; (**e**) Eucalyptus 3 °C·min^−1^.

Concerning the effect of heating rate Figure 3 and Figure 4, and Table 7 show the results obtained on the textural characterization of the solids obtained with olives and eucalyptus samples in pyrolysis at 750 °C with different heating rates.

**Table 7:** Effect of heating rate in the textural characterization of the bioreducers (750 °C).

	Heating Rate (°C·min^−1^)	Real Density (g·cm^−3^)	Micropore Volume (cm^3^·g^−1^)	Micropore Equivalent Surface Area (m^2^·g^−1^)
Olives	20	1.926	0.16	375
	15	1.867	0.15	362
	3	1.871	0.15	363
Eucalyptus	20	1.849	0.20	476
	3	1.830	0.21	501

There is no clear influence of heating rate on the textural properties. Table 7 shows that in the case of olives the real density, micropore volume, and surface area somewhat decrease with the decrease in the heating rate, but in the case of the eucalyptus sample the density also decreases but the micropore volume and surface area somewhat increase. Figure 3 and Figure 4 also show a different effect of heating rate in olive solids and in eucalyptus solids. It has been reported that the longer the char residence time, the greater its surface area [31,32]. This was not the case in this study, which may be due to the fact that 20 °C·min^−1^ is a low enough heating rate under which no further increase in surface area is produced.

If the surface areas of the bioreducers are compared to those presented in the literature, it can be seen that there are great differences. Very low charcoal surface areas have been reported by several authors: Huo, *et al.* [33] reported N_2_ BET area < 9 m^2^·g^−1^ for chars obtained at 900 °C from saw dust and straw, Burhenne, *et al.* [29] reported N_2_ BET area ≈ 1 m^2^·g^−1^ for spruce wood chars obtained at 800 °C, Senneca [34] also reported N_2_ BET areas < 1 m^2^·g^−1^ for char samples obtained from pine wood at 850 °C.

On the contrary, other authors report surface areas in the range of those obtained in this study. Rösler, *et al.* [35] obtained surface areas (measured by CO_2_ adsorption) between 325 and 425 m^2^·g^−1^ for biochars obtained at 750 °C, Burhenne, *et al.* [29] obtained BET areas of 200–400 m^2^·g^−1^ for chars produced at 500 °C, and Carrier, *et al.* [36] reported a N_2_ BET area of 259 m^2^·g^−1^ for char obtained from sugarcane bagasse at 460 °C and 349–452 m^2^·g^−1^ for that same char but after heating it to 800 °C and 900 °C in a N_2_ atmosphere, as well as 441–570 m^2^·g^−1^ after activating the char with steam at 700–900 °C.

The reason for these differences in the surface areas is, most probably, the determination method used. As has been mentioned before, it is difficult to obtain reliable results with N_2_ adsorption methods with carbonaceous materials, since it is carried at −196 °C and at such low temperature, N_2_ has diffusion problems in the narrow micropores and therefore, it may take a long time to reach thermodynamic adsorption equilibrium, it may take (if reached) even weeks. Another reason for erroneous BET area measurements with liquid N_2_ that has been reported in the literature [37], is that there can be pore shrinkage, so that the measured surface areas are apparent, and not real. In this study, in the first place, it was tried to determine the surface areas by liquid N_2_ adsorption, but for most of the bioreducers samples the equilibrium was not reached, even after a week, and in those samples that reached equilibrium, the BET area obtained was very low (7–15 m^2^·g^−1^).

The bioreducers obtained in this study have surface areas comparable to those reported by Carrier, *et al.* [36]. In particular, the eucalyptus derived bioreducers present surface areas (476–501 m^2^·g^−1^) equivalent to those reported by Carrier, *et al.* [36] for sugarcane derived chars activated with steam at 700–900 °C for 1 hour (441–570 m^2^·g^−1^). Therefore, the eucalyptus bioreducers could be used as a sorbent material without needing to be activated. However, it has to be mentioned that the surface area of the eucalyptus bioreducers, the same as that of Carrier, *et al.* [36] activated chars, are well below commercial activated carbon values (BET area ≈ 1000 m^2^·g^−1^).

A comparison between the textural properties of the three commercial reducing agents and of the pyrolysis solids obtained from olives and eucalyptus samples at 750 °C and 3 °C·min^−1^ are presented in Table 8.

**Table 8:** Textural characterization of commercial reducers and bioreducers (750 °C and 3 °C·min^−1^).

	Real Density (g·cm^−3^)	Micropore Volume (cm^3^·g^−1^)	Micropore Equivalent Surface Area (m^2^·g^−1^)
Olives	1.871	0.15	363
Eucalyptus	1.830	0.21	501
Metallurgical coke	1.916	0.01	24
Petroleum coke	1.389	0.07	156
Anthracite	1.793	0.05	122

Concerning real density, Table 8 shows that the bioreducers are comparable to metallurgical coke and anthracite, while petroleum coke has a significantly lower real density. This may be attributed to the lower ash content and the higher micropore volume of petroleum coke.

With respect to surface area, it can be seen that the bioreducers surface areas are much greater than those of the commercial reducers. Therefore, a higher reactivity of the bioreducers compared to the commercial reducers might be expected (this will be discussed in the following section).

**Figure 5:**
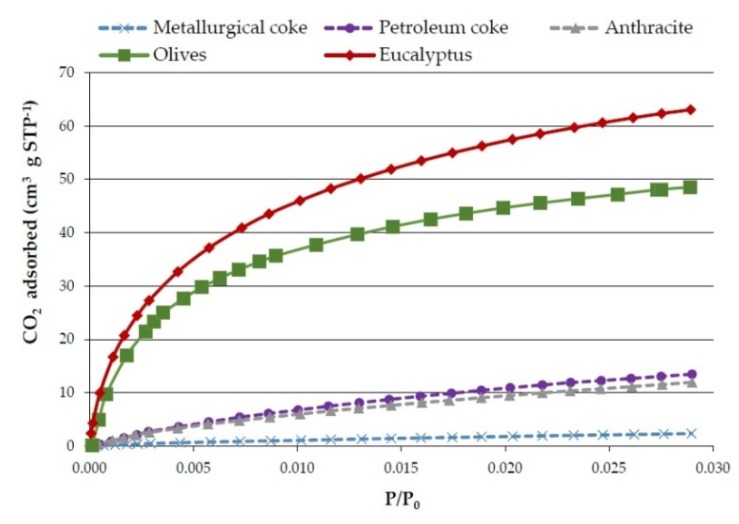
CO_2_ adsorption isotherms of commercial reducers and olives and eucalyptus bioreducers (obtained at 750 °C and 3 °C·min^−1^).

Figure 5 shows the CO_2_ adsorption isotherms of the three commercial reducing agents and of the pyrolysis solids obtained from olives and eucalyptus samples at 750 °C and 3 °C·min^−1^. It has to be mentioned that although it is not clearly visible (due to the scale) the commercial reducers isotherms are also Type I, which is typical of microporous materials. However, as might have been expected, according to the surface area data, the commercial reducers’ curves are much lower than those of the bioreducers.

**Figure 6:**
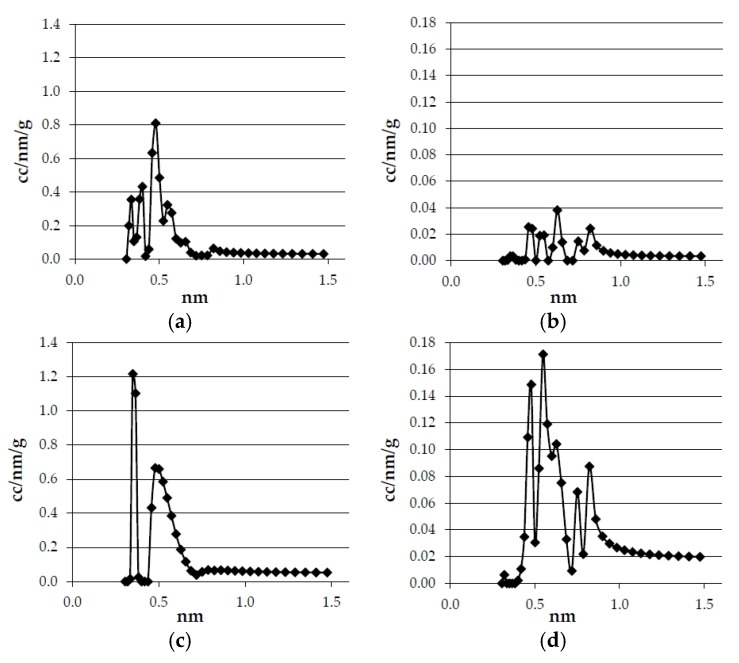

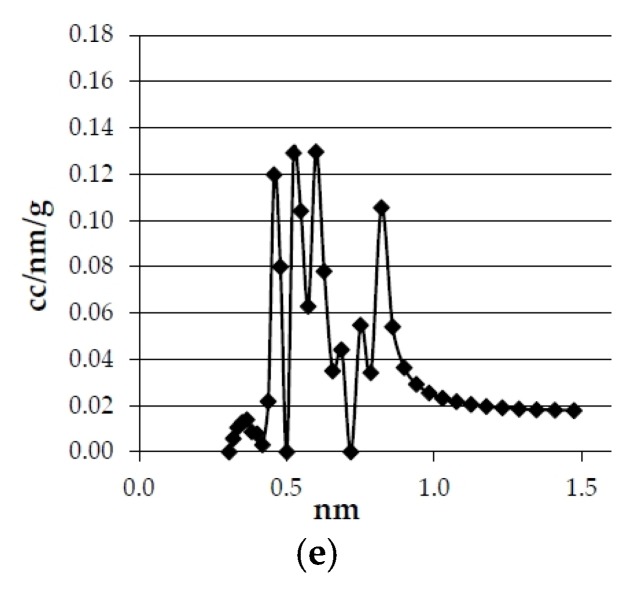
Pore size distribution of commercial reducers and olives and eucalyptus bioreducers (obtained at 750 °C and 3 °C·min^−1^). (**a**) Olives; (**b**) Metallurgical coke; (**c**) Eucalyptus; (**d**) Petroleum coke; (**e**) Anthracite.

The pore size distributions of the bioreducers and the commercial reducing agents calculated by the NL-DFT method are presented in Figure 6. It can be seen that there are important differences between the curves that correspond to the commercial reducers and the pore size distribution of the bioreducers. First of all, the amount of pore of each size is much smaller in the commercial reducers than in the bioreducers. It has to be born in mind that the *y*-axis scale is about 10 times lower in the commercial reducers’ graphs than in the bioreducers’ graphs.

To summarize, the main conclusion concerning the comparison of the bioreducers with the commercial reducing agents is that, although in terms of composition (proximate and ultimate analyses) both types of reducing agents are quite similar, or even better, the bioreducers, in terms of surface area and porosity, are very different, and this brings about, as will be seen in the following section, different reactivity behaviors.

### 3.3. Reactivity

The reactivity experiments carried out were classified in the following three groups:

- Reactivity tests with the three commercial reducers (metallurgical coke, petroleum coke, and anthracite) using 50 g of sample and 1375 mL·min^−1^ of CO_2_ flow.
- Reactivity tests with metallurgical coke using different amounts of sample and two CO_2_ flow ratios (1375 mL·min^−1^ and 750 mL·min^−1^).
- Reactivity tests with the two bioreducers (olives and eucalyptus) using 30 g of sample and 750 mL·min^−1^ of CO_2_ flow.

The reasons for performing these three sets of experiments as well as the results obtained are explained in the following section.

#### 3.3.1. Reactivity Tests with the Commercial Reducers

The objective of this first set of reactivity tests was to determine the reactivity behavior of the reducing agents used and provided by Befesa Zinc Aser S.A. (metallurgical coke, petroleum coke, and anthracite). As has been mentioned in Section 2.2.2 the reactivity tests were performed based on the ASTM D 5341-99 standard but with a smaller amount of sample. In this first set of tests, 50 g of sample and 1375 mL·min^−1^ CO_2_ flow were used, which are proportional to the specifications of the ASTM standard.

The results obtained of both, CRI and R factor, are presented in Table 9. It can be seen that the metallurgical coke and anthracite have similar reactivities according to both, their CRI and R factor, while both parameters are significantly lower for the petroleum coke. Agirre, *et al.* [30] have reported CRI and R values for a typical commercial petroleum coke (13.7% and 16.5%, respectively) which are comparable but somewhat lower than those obtained in this study (16.5% and 20.0%, respectively). Other authors have also observed that petroleum cokes have lower reactivities than metallurgical cokes [38]. It has been reported that this may be because petroleum coke has somewhat more crystalline structures which are less reactive [39].

**Table 9:** Reactivity results of commercial reducing agents (50 g and 1375 mL·min^−1^ CO_2_).

	Metallurgical Coke	Petroleum Coke	Anthracite
CRI (%)	30.9 ± 0.1	16.5 ± 0.2	31.6 ± 0.7
*R* factor (%)	47.4 ± 1.8	20.0 ± 0.1	47.4 ± 0.6

After this first set of reactivity tests, it was attempted to determine the reactivity of the pyrolysis solids. However, due to the lower apparent density of these solids (0.45 g cm^−3^ for olives and 0.42 g·cm^−3^ for eucalyptus, compared to 1.11 for metallurgical coke, 0.72 for petroleum coke and 1.31 for anthracite), it was not possible to introduce 50 g of sample in the tubular reactor. Therefore, it was decided to carry out some experiments to investigate if the amount of sample used could have an influence on the results obtained on the reactivity tests. The results are presented in the following section.

#### 3.3.2. Reactivity Tests of the Metallurgical Coke with Different Amounts of Sample and Different CO_2_ Flows

The CRI and *R* factor obtained with the metallurgical coke using different amounts of sample and different CO^2^ flows are presented in Table 10.

**Table 10:** Reactivity results of metallurgical coke.

	1375 mL·min^−1^			750 mL·min^−1^
	50 g	30 g	15 g	30 g
CRI (%)	30.9 ± 0.1	43.5 ± 1.3	41.0 ± 2.0	34.5 ± 0.5
*R* factor (%)	47.4 ± 1.8	27.0 ± 0.4	9.8 ± 0.1	45.9 ± 1.2

The reactivity results obtained with 1375 mL·min^−1^ CO_2_ flow and with different amounts of sample show that the CRI index significantly increases when the amount of sample is reduced from 50 g to 30 g, but no further increase is observed from 30 g to 15 g. The explanation of this fact may be the following: the greater the amount of sample, the larger is the height occupied by it inside the tubular reactor; the CO_2_ stream is fed into the reactor through the bottom, therefore it starts reacting with the coke, and as it goes up through the char bed it is impoverished in CO_2_, leading to a lower reaction rate at the upper part of the reactor. This effect is not observed from the 15 g to the 30 g tests probably because with smaller column heights there is CO_2_ enough all through the column both with the 15 and the 30 g samples and therefore, the negative effect of increasing the amount of sample is not observed in this range.

Regarding the *R* factor, it clearly decreases when the amount of sample is reduced in the whole mass sample range. This was obviously the expected trend, since as less sample is used, less total CO is produced, and consequently, for the same CO_2_ flow, the CO/CO_2_ ratio is lower and therefore, the R factor as well.

Comparing the results obtained with 30 g of sample at the two different CO_2_ flows, it was observed that the CRI decreases while the *R* factor increases as the CO_2_ flow decreases. This was to be expected since, if there is less CO_2_ available for the same amount of sample, on the one hand, less coke reacts and therefore, the weight loss is smaller, and consequently the CRI decreases. On the other hand, since the CO_2_ concentration is much lower, even though less CO has been produced, the CO/CO_2_ ratio increases, and consequently, the R factor increases.

If the results obtained with 30 g and 750 mL·min^−1^ are compared with those obtained with 50 g and 1375 mL·min^−1^, it can be seen that they are quite similar, which is attributed to the fact that the amount of sample/CO_2_ flow ratio used in these two tests is quite similar (≈26).

Therefore, it has been demonstrated that in the reactivity tests the amount of sample used, plays a very important role, but that if the amount of sample/CO_2_ flow ratio is kept constant, the influence of the amount of sample can be neglected.

Therefore, the reactivity tests of the bioreducers, which are presented in the following section, were carried out with 30 g of sample and 750 mL·min^−1^ CO_2_ flow, so that they could be compared with the reactivity tests of the commercial reducers carried out with 50 g and 1375 mL·min^−1^ CO_2_.

#### 3.3.3. Reactivity Tests with the Bioreducers

The results of the reactivity tests of the pyrolysis solids (olives and eucalyptus) carried out using 30 g and 750 mL·min^−1^ CO_2_ are included in Table 11. It has to be mentioned that the error of these measurements could not be determined in all cases due to the shortage of sample which made it impossible to repeat some of the tests. In those cases in which the errors were determined, the CRI error was in the range 1–3 and the *R* factor error in the range 2–4.

**Table 11:** Reactivity results of pyrolysis bioreducers (750 °C) (30 g and 750 mL·min^−1^ CO_2_).

	Olives			Eucalyptus
	20 °C·min^−1^	15 °C·min^−1^	3 °C·min^−1^	3 °C·min^−1^
CRI (%)	87.8	86.8	86.1	93.7
*R* factor (%)	81.3	82.4	74.4	76.1

It can be seen that the reactivity of the bioreducers is very high. In the case of the olives sample, the CRI values are around 87% therefore only 13% of the initial mass has not reacted in the reactivity test. Taking into account that the ash contents of the olive derived bioreducers is close to 10% (Table 5) it can be concluded that the sample has almost completely reacted with CO_2_. The same happens in the case of the eucalyptus derived bioreducer: the CRI value is as high as 94% when it has an ash content of 5.4%. Therefore, the values in Table 11 can be considered to be the maximum achievable reactivities. Burhenne, *et al.* [29] also obtained almost 100% reactivity of spruce wood derived char in tests carried out both in TGA at 800 °C for 3–5 h and in a fixed bed reactor at 800 °C for 30 min. For this reason, a discussion of the influence of the heating rate or type of sample on the reactivity of the bioreducers obtained in this study does not deserve attention.

Comparing Table 9 and Table 11 it can be seen that the reactivity of bioreducers is far higher than that of the commercial reducers. This may be attributed to the fact that the surface areas of the commercial reducers are much lower (24–156 m^2^·g^−1^) than those of the bioreducers derived from olives and eucalyptus samples (363–501 m^2^·g^−1^). Huo, *et al.* [33] also obtained much higher reactivities of bioreducers than of petroleum coke or anthracite, and they also reported that there was a relationship between reactivity and surface area.

On the contrary, bioreducers obtained by pyrolysis of fruit tree cuttings at 900 °C by Agirre, *et al.* [30], have CRI values in the range of 20%–50% and *R* factor 35%–70%, both values much lower than those obtained in this study, and comparable to those of commercial reducing agents. There are several reasons to explain these differences: (1) on the one hand, the equipment used for reactivity measurements by Agirre, *et al.*, is very different from the one used in this study; it is a horizontal tubular furnace and the sample is placed in a crucible inside the reactor, therefore the CO_2_ stream is only in contact with the upper surface of the sample, while in this study a vertical reactor is used, and therefore the CO_2_ stream goes through the whole bioreducer bed; (2) the reaction time once reached at the reaction temperature (1000 °C) used by Agirre, *et al.*, was only 15 min instead of 2 h which is the time specified in the ASTM D 5341-99 and the one used in the reactivity tests of this study; (3) the particle size used by Agirre, *et al.*, (4–5 cm) was larger than that used in this study (1 cm); (4) the type of original biomass from which bioreducers were derived was different (fruit tree cuttings *vs.* olive tree cuttings and eucalyptus); this fact, although less influential than the previous ones, may also be of importance.

In view of the characteristics of the olive and eucalyptus derived bioreducers and in order to analyze their suitability for metallurgical processes, it is worth mentioning that there are three roles that coke plays in metallurgical processes: (1) it has to provide heat or energy; (2) it has to provide a reducing atmosphere by means of the reaction: $\text{C}+{\text{ CO}}_{2}\;↔2\text{ CO}$; (3) it has to act as a support medium for the burden if the process is carried out in a blast furnace.

The first two requirements are well fulfilled by the bioreducers obtained in this study; they have rather high heating values (≈30 MJ·kg^−1^) and they are fully reactive. However, bioreducers cannot fulfill the third requirement, and therefore, due to their missing strength and high reactivity, cannot be used in blast furnaces as support medium, but they can be used tuyére-injected in the lower part of the blast furnaces to substitute typically used fossil fuels (pulverized carbon, oils, natural gas). On the other hand, bioreducers can also be used in the non-ferrous metal industry since no mechanical strength is required, because rotary kilns, where the burden is mixed and rotates with the coke, are frequently used.

Nevertheless, the final decision to determine the suitability of bioreducers for the non-ferrous metallurgical industry depends on the specific characteristics of the process itself. Metal reduction experiments should be carried out with the bioreducers in order to determine if the high reactivities could be a handicap for the process, but such kind of experiments were beyond the scope of this paper. However, it has been reported in the literature, experiments on the reduction of electric arc furnace dusts carried out with charcoal which show that with bioreducers higher zinc volatilization and iron oxide reduction can be achieved in comparison to those obtained with the fossil cokes normally used in industry [15].

## 4. Conclusions

As far as proximate and ultimate analyses are concerned, olives and eucalyptus derived reducing agents (bioreducers) are of better quality than typical commercial reducing agents used in non-ferrous processes (metallurgical coke, petroleum coke, anthracite), since the former have lower ash and sulfur contents.

The surface area and porosity of the bioreducers obtained in this study (363–501 m^2^·g^−1^ and 0.15–0.21 cm^3^·g^−1^, respectively) are much higher than those of typical commercial reducing agents used in metallurgical processes in rotary kilns (24–156 m^2^·g^−1^ and 0.01–0.07 cm^3^·g^−1^, respectively).

The bioreducers reactivity measurements with CO_2_ are very much conditioned by the experimental procedure used. If the ASTM D 5314-99 standard procedure cannot be strictly followed due to sample shortage, a mass sample/CO_2_ flow ratio equivalent to that of the ASTM standard should be used.

The bioreducers obtained in this study have no mechanical strength and present extremely high reactivity with CO_2_ (close to 100%). Therefore, they cannot be used as top burden in blast furnaces, but they can be used as fuel and reducing agent either tuyére-injected at the lower part of the blast furnace or in non-ferrous metallurgical processes where no mechanical strength is needed, such as in rotary kilns.
